# The impact of early vs. delayed surgery on outcomes in cervical spinal cord injury without fracture or dislocation

**DOI:** 10.3389/fsurg.2025.1619141

**Published:** 2025-08-13

**Authors:** Sirui Xiao, Hui Yan, Beixi Bao, Yuxuan Wu, Xiaokang Cheng, Chunyang Xu, Jiaguang Tang

**Affiliations:** Department of Orthopedics, Beijing Tongren Hospital, Capital Medical University, Beijing, China

**Keywords:** cervical spinal cord injury without fracture or dislocation, early surgery, delayed surgery, anterior cervical discectomy and fusion (ACDF), posterior cervical surgery, JOA scores, ASIA score

## Abstract

**Objective:**

This study aims to retrospectively analyze 104 patients diagnosed with cervical spinal cord injury without fracture or dislocation (CSCIwoFD) who underwent surgical treatment, in order to compare the effects of early vs. delayed surgical intervention on neurological functional recovery.

**Methods:**

Patients diagnosed with CSCIwoFD and treated surgically at our institution between August 2020 and January 2023 were retrospectively reviewed. Based on the time interval from injury to surgery, patients were categorized into two groups: early surgery group (Group A) and delayed surgery group (Group B). Neurological function was assessed using the Japanese Orthopaedic Association (JOA) score, the American Spinal Injury Association motor score (AMS), and sensory score (ASS). Improvement rates were calculated as the JOA recovery rate (RR), AMS recovery rate (AMSRR), and ASS recovery rate (ASSRR). Pearson correlation analyses were performed using R software to determine the linear relationships between postoperative neurological outcomes and imaging parameters, including maximum canal compromise (MCC), maximum spinal cord compression (MSCC), developmental spinal canal stenosis, and ossification of the posterior longitudinal ligament (OPLL), as well as with postoperative complications. Intergroup comparisons were also made regarding hospitalization duration, time to return to work post-discharge, in-hospital treatment costs, and patient satisfaction 2 years after treatment.

**Results:**

MSCC showed a significant positive correlation with postoperative neurological recovery metrics including RR, JOA, AMS, and AMSRR, while its correlation with ASSRR was weaker. No significant associations were observed between MSCC and patient age, sex, or surgical approach; however, a mild positive correlation with surgical timing was identified. Compared to the delayed surgery group, the early surgery group exhibited longer operative duration, greater intraoperative blood loss, and higher postoperative drainage volume, but there are no significant difference in the incidence of complications (*P* > 0.05). There were also no significant differences between groups in terms of the number of fused segments, bone fusion rate at 6 months, patient satisfaction at 2 years, length of hospital stay, time to return to work, or hospitalization costs (*P* > 0.05). Imaging parameters revealed higher MCC and MSCC values in the early surgery group, but only the difference in MSCC reached statistical significance (*P* < 0.05).

**Efficacy evaluation:**

At admission, there were no a significant differences in JOA, AMS, or ASS scores between the groups. Postoperatively, both groups showed improvements in JOA, ASS, and AMS scores, with significantly better outcomes in the early surgery group. Intergroup comparisons at 6 months, 1 year, and 2 years postoperatively showed statistically significant differences in JOA and AMS scores (*P* < 0.05), and significant differences in ASS scores at 1 and 2 years postoperatively (*P* < 0.05). Notably, the 2-year postoperative JOA score difference between groups was 2.71 points, exceeding the minimal clinically important difference (MCID) threshold. For recovery rates (RR, ASSRR, AMSRR), statistically significant differences were found between groups at both 1 and 2 years postoperatively (*P* < 0.05). In the early surgery group, Pearson analysis indicated that MSCC was positively correlated with RR at 3 months (*γ* = 0.527, *P* < 0.05) and AMSRR at 3 months (*γ* = 0.277, *P* < 0.05).

**Conclusion:**

Both early and delayed surgical interventions can improve spinal cord function in patients with CSCIwoFD; however, early surgery is associated with better neurological recovery. Among imaging predictors, MSCC is particularly effective in forecasting motor recovery in early surgery patients. When determining the optimal timing for surgery, clinicians should consider individual comorbidities and the severity of spinal cord injury. Under conditions of stabilized traumatic stress response, surgical intervention within 7 days of injury is recommended to maximize neurological recovery and prognosis.

## 1 Introduction

In recent years, with societal development and population aging, the incidence of cervical spinal cord injury (SCI) has continued to rise. As a condition that may result in lifelong disability and significant healthcare resource utilization, cervical SCI remains a major concern for spine surgeons. Cervical spinal cord injury without fracture and dislocation (CSCIwoFD), also referred to as spinal cord injury without radiographic abnormality (SCIWORA), is a specific subtype of incomplete cervical SCI commonly encountered in clinical practice. Due to cervical degenerative changes, the true incidence of CSCIwoFD in adults is substantially underestimated despite its high prevalence (1, 2). These patients typically present with a clear history of cervical trauma accompanied by spinal cord dysfunction, manifesting as limb weakness or paralysis, numbness, and impaired bladder and bowel control. Magnetic resonance imaging (MRI) often reveals spinal cord compression and damage to intervertebral discs and surrounding soft tissues, while x-ray and CT imaging fail to demonstrate vertebral fractures or cervical alignment abnormalities.

Consistent with previous studies (3), clinical research at our institution has demonstrated that anterior cervical discectomy and fusion (ACDF) offers superior neurological recovery compared to conservative treatment in CSCIwoFD patients with MRI evidence of spinal cord compression. However, the optimal surgical timing for incomplete cervical SCI, particularly CSCIwoFD, remains controversial. Prior studies and meta-analyses have primarily focused on comparing outcomes of ultra-early (within 24 h) or early (within 72 h) surgical interventions (4, 5), suggesting that earlier decompression may yield greater neurological benefits. Nevertheless, the efficacy of delayed surgery in subacute or chronic CSCIwoFD cases has not been clearly established, and the underlying injury mechanisms remain debated. These patients often present with concomitant polytrauma, and some studies have reported that delayed surgical intervention may reduce mortality and contribute to partial neurological recovery (6).

In light of these considerations, we retrospectively analyzed patients diagnosed with CSCIwoFD at our institution who underwent surgical treatment. Based on the timing of surgical intervention, patients were categorized into early and delayed surgery groups, and their postoperative neurological recovery outcomes were compared.

## 2 Materials and methods

### 2.1 General information

A total of 104 patients diagnosed with cervical spinal cord injury without fracture or dislocation (CSCIwoFD) and treated surgically at our hospital between August 2020 and January 2023 were retrospectively analyzed. The time from injury to hospital admission ranged from 6 h to 25 days. Based on the timing of surgery, patients were divided into two groups: Group A (early surgery, ≤7 days) and Group B (delayed surgery, >7 days). Patients were randomly assigned to either the early surgery group (Group A) or the delayed surgery group (Group B). Randomization was performed using a computer-generated random number table to ensure allocation concealment. The allocation sequence was managed by an independent researcher who was not involved in the clinical treatment process. After completing follow-up, the group distribution was as follows:

Group A (Early Surgery Group): 54 patients, including 39 males and 15 females, aged 25–69 years (mean age: 49.50 ± 12.13 years). Mechanisms of injury included traffic accidents (*n* = 21), falls from standing height (*n* = 18), falls from height (*n* = 8), and other types of trauma (*n* = 7).

Group B (Delayed Surgery Group): 50 patients, including 34 males and 16 females, aged 24–72 years (mean age: 54.55 ± 9.28 years). Injury mechanisms included traffic accidents (*n* = 19), falls from standing height (*n* = 14), falls from height (*n* = 9), and other traumatic events (*n* = 8).

This study was approved by the Medical Ethics Committee of Beijing Tongren Hospital, Capital Medical University. The clinical trial registration number is ChiCTR1900025109, and the date of registration is Aug-11-2019.This study was retrospective in nature, and both the patients and their families were informed of the differences in the treatment protocols, provided their consent to participate, and signed an agreement. All of the patients were followed up for at least 2 years via phone calls or scheduled outpatient visits. There were no statistically significant differences between the two groups in terms of age, sex, or injury mechanism (*P* > 0.05), indicating comparability. Detailed data are presented in Table 1.

**Table 1:** Baseline characteristics of the study participants.

Variable		Early surgery group Group A	Delayed surgery group Group B	*P* score
Cases		54	50	
Age (years)		49.50 ± 12.13	54.55 ± 9.28	0.682
Gender	Male	39	34	0.418
	Female	15	16	
Reason (*n*)	Fall injury	18	14	0.563
	Fall injury from high	8	9	
	Traffic	21	19	
	Others	7	8	
Imaging indicators				
Maximum canal compromise (MCC) %		41.45 ± 12.55	39.87 ± 10.62	0.376
Maximum spinal cord compression (MSCC) %		21.28 ± 8.63	18.24 ± 9.29	0.027
Surgical indicators				
Number of fused segments	1	29	20	0.377
	2	19	22	
	3	5	6	
	4	1	2	
Surgical method (*n*)	ACDF	48	42	0.31
	Posterior	6	8	
Duration of surgery (min)		149.79 ± 43.33	144.95 ± 39.82	0.202
Intraoperative blood loss (ml)		484.64 ± 35.70	470.97 ± 32.38	0.419
Postoperative drainage volume (ml)		117.73 ± 19.92	105.25 ± 15.88	0.399
Bone fusion at 6 months		54	50	1.00
Complications Incidence *n* (%)				
Infection		3	1	0.366
Hemorrhage		2	2	
Liquefactive necrosis of the wound		0	0	
Cerebrospinal fluid (CSF) leakage		2	1	
Urinary tract infection		10	7	
Spinal cord neurological dysfunction		2	1	
Pneumonia		7	6	

### 2.2 Inclusion and exclusion criteria

Inclusion Criteria: (1) A clear history of cervical trauma, such as falls, high-energy impact from height, or motor vehicle accidents; (2) Cervical spine MRI revealing varying degrees of degenerative changes and abnormal signal intensity at the lesion level, with no evidence of fracture or dislocation on cervical x-ray or CT imaging; (3) Clinical manifestations and signs of spinal cord injury corresponding to the affected cervical segment, such as hypoesthesia, hyperesthesia, or motor dysfunction; (4) A minimum outpatient follow-up period of 2 years; (5) No previous history of central nervous system or psychiatric disorders; (6) No history of spinal surgery.

Exclusion Criteria: (1) Presence of cerebrovascular or thoracic diseases that could affect spinal cord or neurological function before or after treatment, such as cerebral infarction or intracranial hemorrhage; (2) Fractures or traumatic injuries involving the cervical spine or other skeletal regions; (3) Severe osteoporosis or other conditions rendering the patient unfit for surgical intervention; (4) Psychiatric disorders or cognitive impairment; (5) Pathological spinal lesions; (6) Patients who were lost to follow-up or died during postoperative outpatient follow-up.

### 2.3 Imaging examinations

All patients routinely underwent cervical spine x-ray, CT, and MRI examinations. None showed evidence of cervical vertebral fractures or dislocations. Developmental cervical spinal stenosis (DCSS), ossification of the posterior longitudinal ligament (OPLL), and ligamentum flavum ossification were assessed using x-ray and CT imaging. MRI was utilized to evaluate the degree of spinal cord compression and to assess intervertebral disc herniation and damage to the posterior longitudinal ligament complex. Based on the method described by Fehlings et al. (7), the following sagittal diameters were measured: the sagittal diameter of the spinal canal at the most stenotic level (Di), the sagittal diameter of the spinal cord at the point of maximum compression (di), as well as the sagittal diameters of the spinal canal (Da, Db) and the spinal cord (da, db) at one segment above and below the injury level, respectively. Using these measurements, the maximum canal compromise (MCC) and maximum spinal cord compression (MSCC) were calculated as follows:

$$\mathrm{MCC}\;=\;\left[1-\frac{2\times \mathrm{Di}}{\left(\mathrm{Da}\;+\;\mathrm{Db}\right)}\right]\;\times \;100\text{\%}$$

$$\mathrm{MSCC}\;=\;\left[1-\frac{2\times \mathrm{di}}{\left(\mathrm{da}\;+\;\mathrm{db}\right)}\right]\;\times \;100\text{\%}$$

### 2.4 Surgical procedures

Upon admission, all patients received cervical immobilization with a neck brace, continuous electrocardiographic monitoring, oxygen supplementation, and nutritional support to stabilize vital signs. While awaiting completion of preoperative imaging and assessments, the following preoperative treatments were administered:

High-dose corticosteroid pulse therapy: 500 mg of methylprednisolone sodium succinate mixed with 250 ml of normal saline, administered via intravenous infusion. Dehydration therapy: 100 ml of mannitol administered intravenously. Gastroprotective therapy: 40 mg of omeprazole sodium mixed with 100 ml of normal saline, administered intravenously.

**Figure 1:**
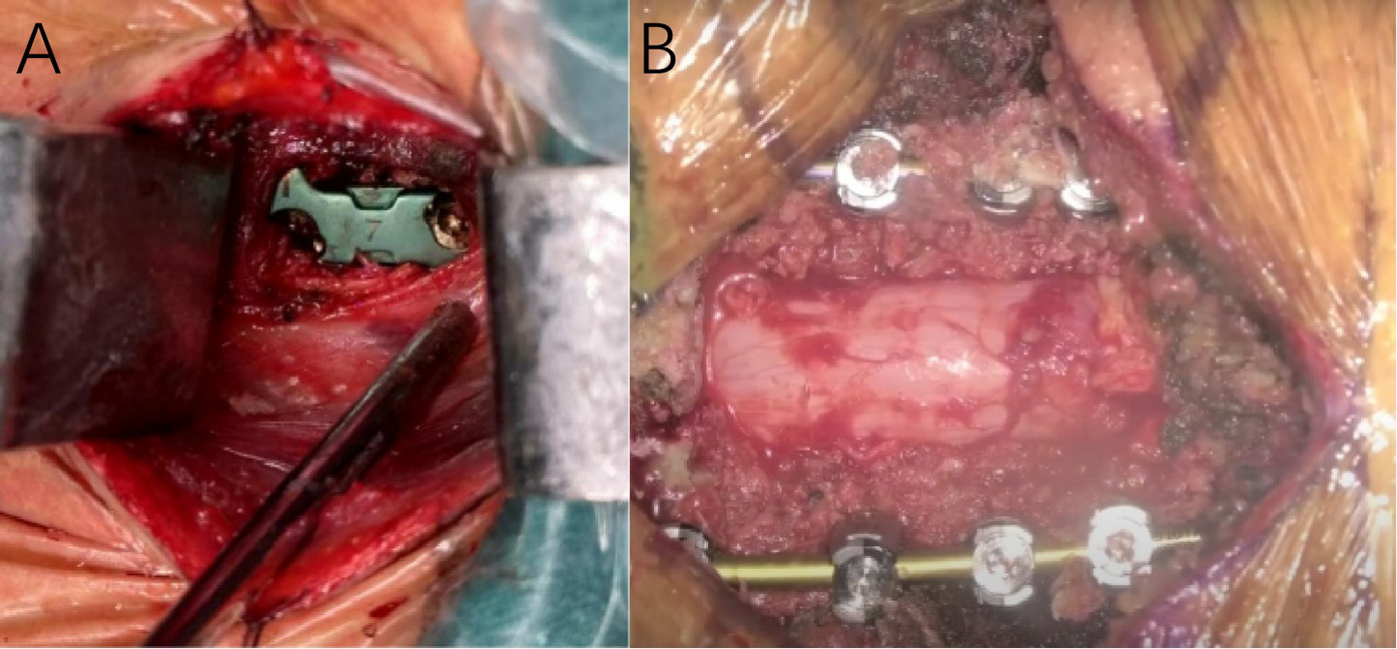
Intraoperative image **(A)** showing the outcome following placement of an interbody fusion device during anterior cervical discectomy and fusion (ACDF). Intraoperative image **(B)** showing the outcome following posterior cervical decompression and internal fixation surgery.

**Figure 2:**
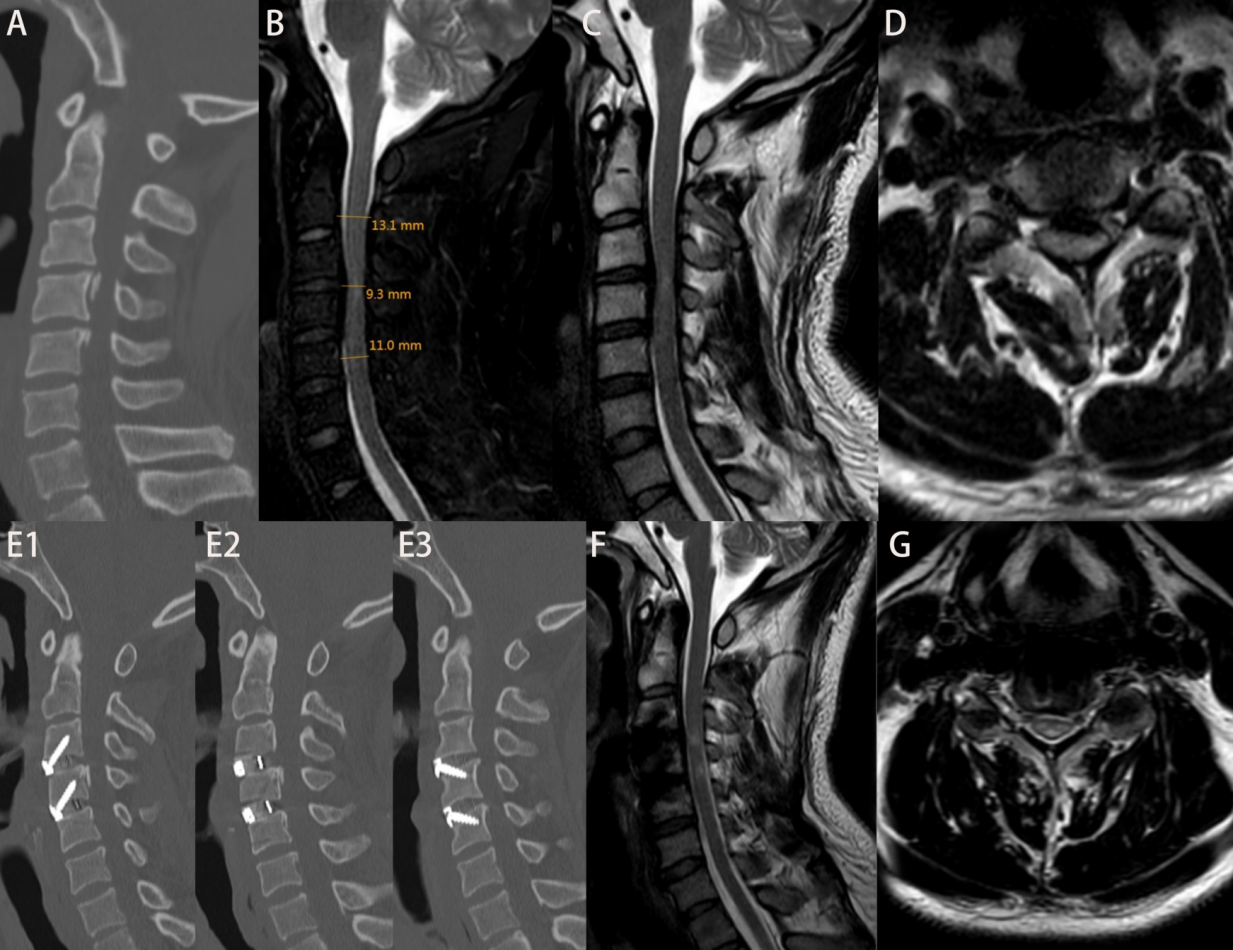
Patient Ma, admitted 6 h after a fall, with an initial JOA score of 9. **(A)** Admission cervical CT scan showing no evidence of fracture or dislocation. **(B)** Measurement of di, da, db, and MSCC values; MSCC calculated to be 22.82%. **(C,D)** Preoperative cervical MRI showing significant spinal cord compression. On the second day post-admission, the patient underwent ACDF at C3–4 and C4–5 levels. The patient was discharged on postoperative day 10. Figures E1–E3: cervical CT at 6 months postoperatively showing good positioning of screws and interbody fusion device. **(F,G)** Cervical MRI at 6 months postoperatively showing decompression of the spinal cord. At 2-year follow-up, the JOA score was 16.

**Figure 3:**
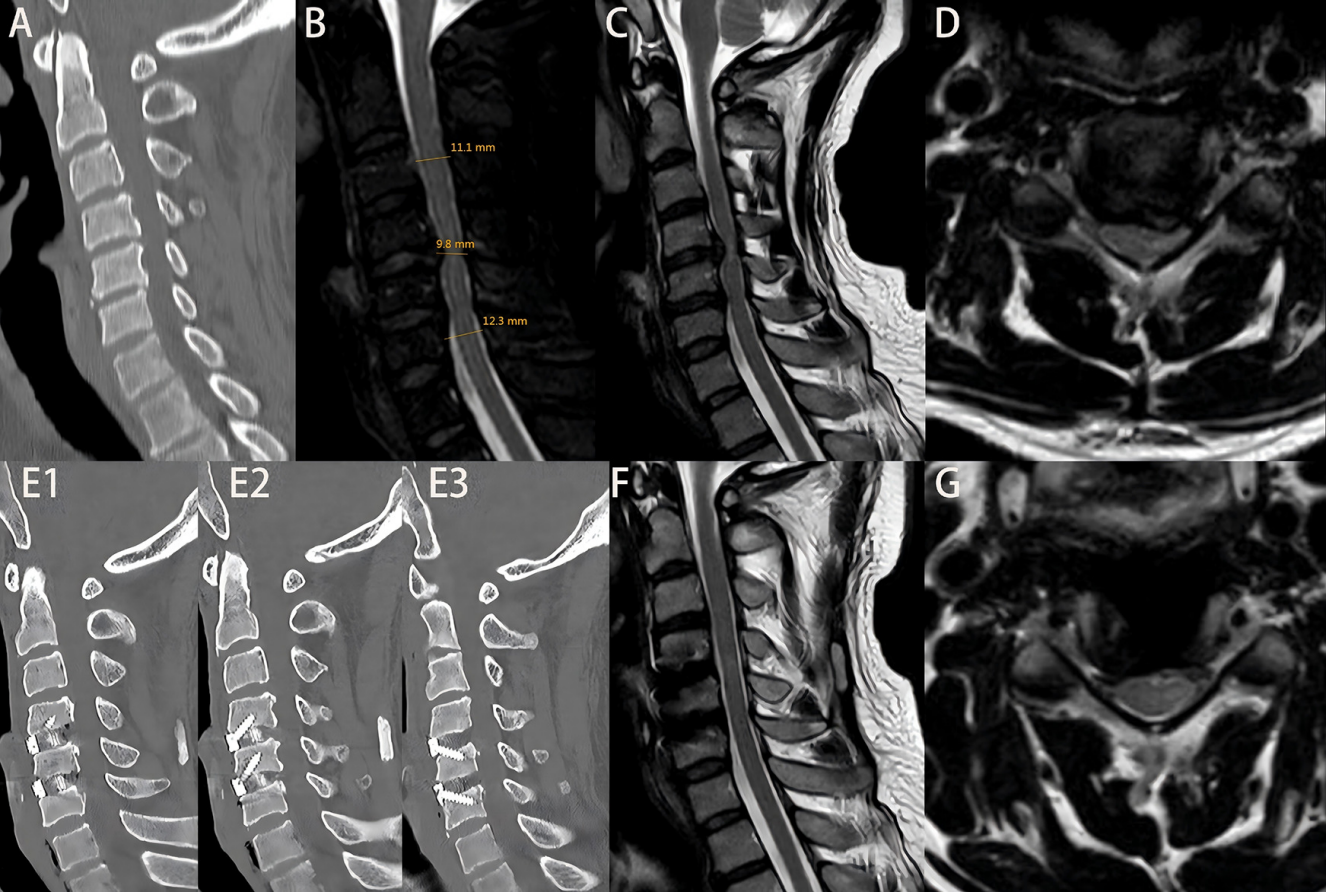
Patient Zhao, admitted 10 days after a traumatic injury, with an initial JOA score of 8. **(A)** Admission cervical CT scan showing no fracture or dislocation. **(B)** Measurement of di, da, db, and MSCC values; MSCC calculated to be 16.24%. **(C,D)** Preoperative cervical MRI showing significant spinal cord compression. On the second day after admission, the patient underwent ACDF at C3–4 and C4–5 levels. The patient was discharged on postoperative day 3. Figures E1–E3: cervical CT at 6 months postoperatively showing proper positioning of screws and interbody fusion device. **(F,G)** Cervical MRI at 6 months postoperatively showing relief of spinal cord compression. At 2-year follow-up, the JOA score was 14.

The surgical approach was determined based on the patient's cervical MRI findings. For patients with localized ventral spinal cord compression—such as disc herniation or ossification of the posterior longitudinal ligament (OPLL)—and involvement of one to two degenerative cervical segments, anterior cervical discectomy and fusion (ACDF) was performed. For those with multilevel degenerative disease or extensive ligamentous ossification, posterior cervical procedures were employed, including posterior cervical laminectomy with internal fixation or laminoplasty for spinal canal expansion. Figure 1 illustrates the intraoperative procedures and outcomes of both anterior and posterior cervical surgeries performed at our institution. All operations were performed or supervised by the same senior spinal surgeon in our hospital. A single cervical wound drainage tube was placed in each case. Postoperatively, patients continued to wear a cervical collar for 2–3 months and received adjunctive treatments, including neurotrophic agents, low-dose corticosteroids, mannitol, gastroprotective agents, and rehabilitation training. Figures 2, 3 presents preoperative and follow-up imaging results of representative cases treated with different surgical approaches and at different time intervals.

### 2.5 Evaluation criteria

#### 2.5.1 General indicators

Length of hospital stay, time to return to work post-discharge, total hospitalization cost, and patient satisfaction at 2 years after treatment.

#### 2.5.2 Surgical indicators

Surgical approach, number of fused segments, operative duration, intraoperative blood loss, postoperative drainage volume, and bone fusion rate at 6 months postoperatively.

#### 2.5.3 Efficacy indicators

Neurological function was assessed at admission and at 3 months, 6 months, 1 year, and 2 years after surgery. The Japanese Orthopaedic Association (JOA) scoring system for cervical myelopathy was used (8), evaluating: Upper extremity motor function (4 points), Lower extremity motor function (4 points), Sensory function of upper/lower extremities and trunk (6 points), Bladder function (3 points). The JOA recovery rate (RR) was calculated using the formula proposed by Hirabayashi et al. (9):

$$\mathrm{RR}\;=\;\left[\frac{\left(\mathrm{JOA}\;\mathrm{score}\;\mathrm{at}\;\mathrm{the}\;\mathrm{last}\;\mathrm{follow}\;\mathrm{up}-\mathrm{JOA}\;\mathrm{score}\;\mathrm{at}\;\mathrm{admission}\right)}{\left(17-\mathrm{JOA}\;\mathrm{score}\;\mathrm{at}\;\mathrm{admission}\right)}\right]\;\times \;100\text{\%}$$

Neurological function was also assessed using the American Spinal Injury Association (ASIA) standards (10), including: ASIA Motor Score (AMS), ASIA Sensory Score (ASS). Neurological improvement rates were calculated as follows:

the ASIA Motor Score Improvement Rate(AMSRR):

$$\mathrm{AMSRR}\;=\;\left[\frac{\left(\mathrm{Posttreatment}\;\mathrm{ASIA}\;\mathrm{motor}\;\mathrm{score}-\mathrm{Pretreatment}\;\mathrm{ASIA}\;\mathrm{motor}\;\mathrm{score}\right)}{\left(100\;-\;\mathrm{Pretreatment}\;\mathrm{ASIA}\;\mathrm{motor}\;\mathrm{score}\right)}\right]\;\times \;100\text{\%}$$

and the ASIA sensory score improvement rate(ASSRR):

$$\mathrm{ASSRR}\;=\;\left[\frac{\left(\mathrm{Posttreatment}\;\mathrm{ASIA}\;\mathrm{sensory}\;\mathrm{score}-\mathrm{Pretreatment}\;\mathrm{ASIA}\;\mathrm{sensory}\;\mathrm{score}\right)}{\left(112\;-\mathrm{Pretreatment}\;\mathrm{ASIA}\;\mathrm{sensory}\;\mathrm{score}\right)}\right]\;\times \;100\text{\%}$$

### 2.6 Statistical analysis

Initial correlation analysis of factors associated with improvements in ASIA motor scores, ASIA sensory scores, and JOA recovery rates was conducted using the R statistical software package, applying linear correlation methods. Subsequent statistical analyses were performed using SPSS version 27.0. For continuous variables conforming to a normal distribution, data were expressed as mean ± standard deviation (x¯ ± s). Independent sample *t*-tests or repeated measures ANOVA were used for intergroup comparisons. Categorical variables were compared using the chi-square (*χ*²) test. A *P*-value < 0.05 was considered statistically significant.

## 3 Results

### 3.1 Correlation analysis

**Figure 4:**
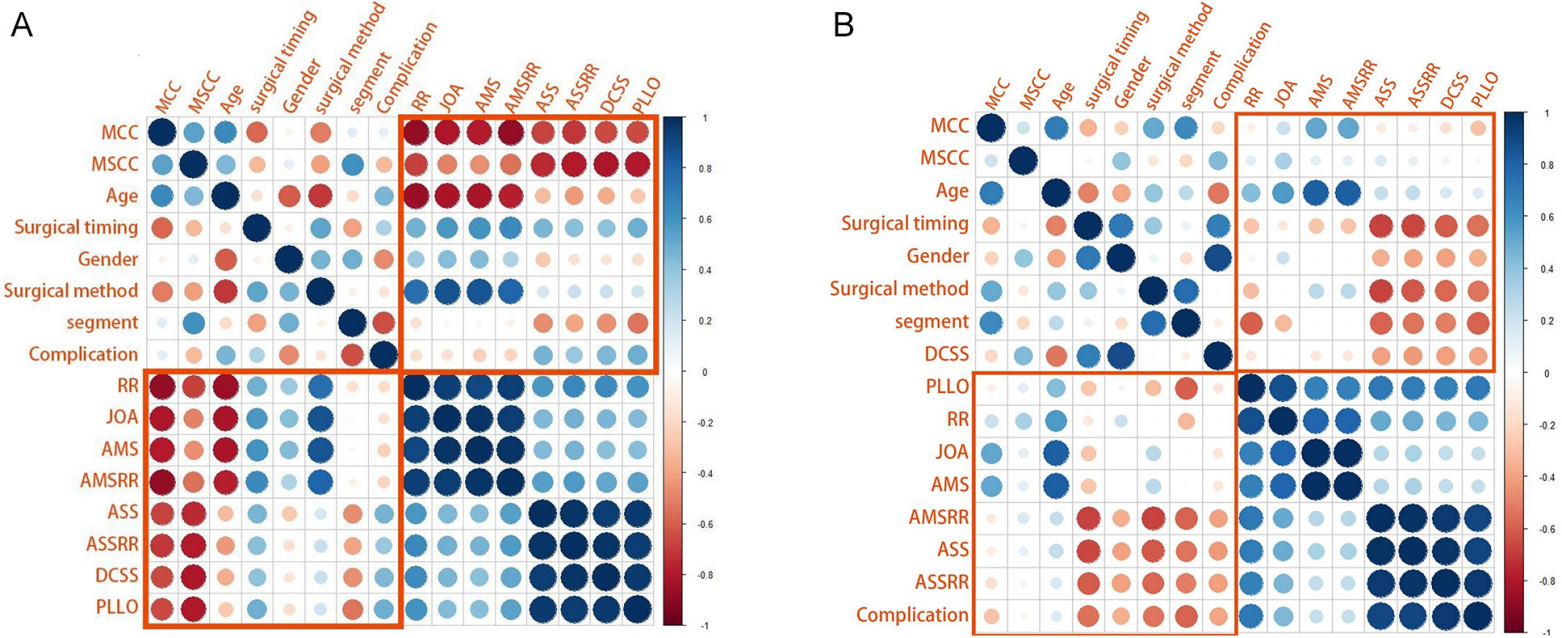
**(A, B)** Show correlation matrices generated using R software for the early and delayed surgery groups, respectively, illustrating relationships between clinical variables. It is used to display the correlation coefficients between variables (ranging from −1 to +1). In the figure: red indicates negative correlation (the closer the value is to −1, the darker the color), blue indicates positive correlation (the closer the value is to +1, the darker the color), and the darkness of the color and the size of the circles represent the strength of the correlation.

**Figure 5:**
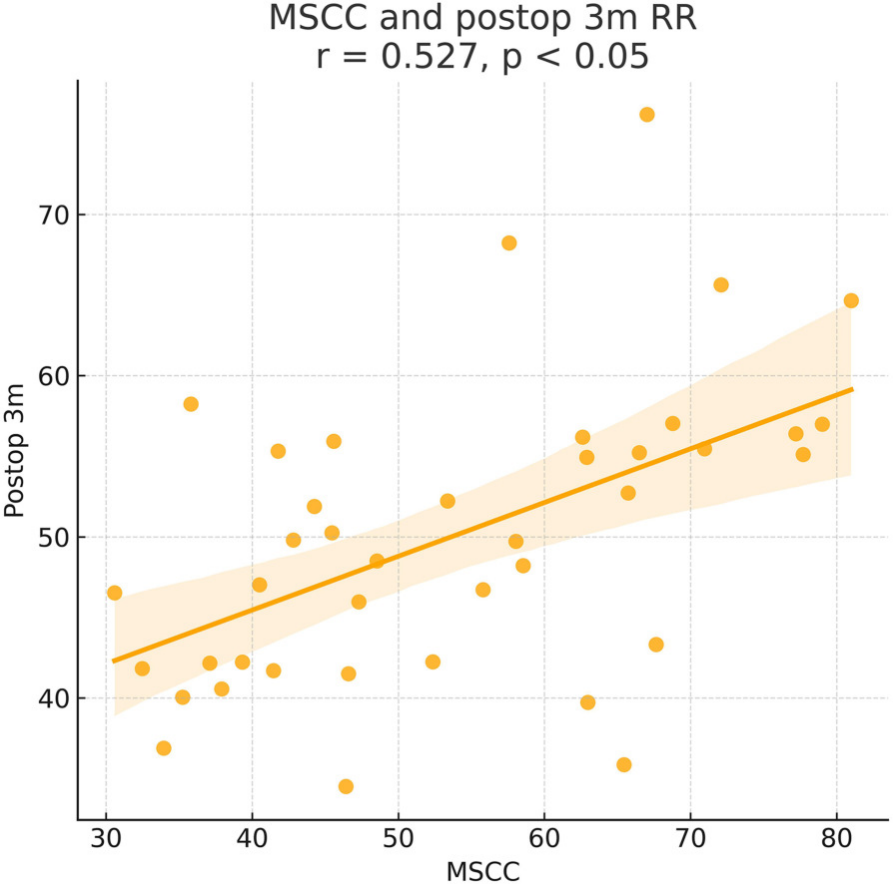
Pearson correlation analysis indicated a significant positive correlation between MSCC and postoperative 3-month recovery rate (RR) (*γ* = 0.527, *P* < 0.05).

**Figure 6:**
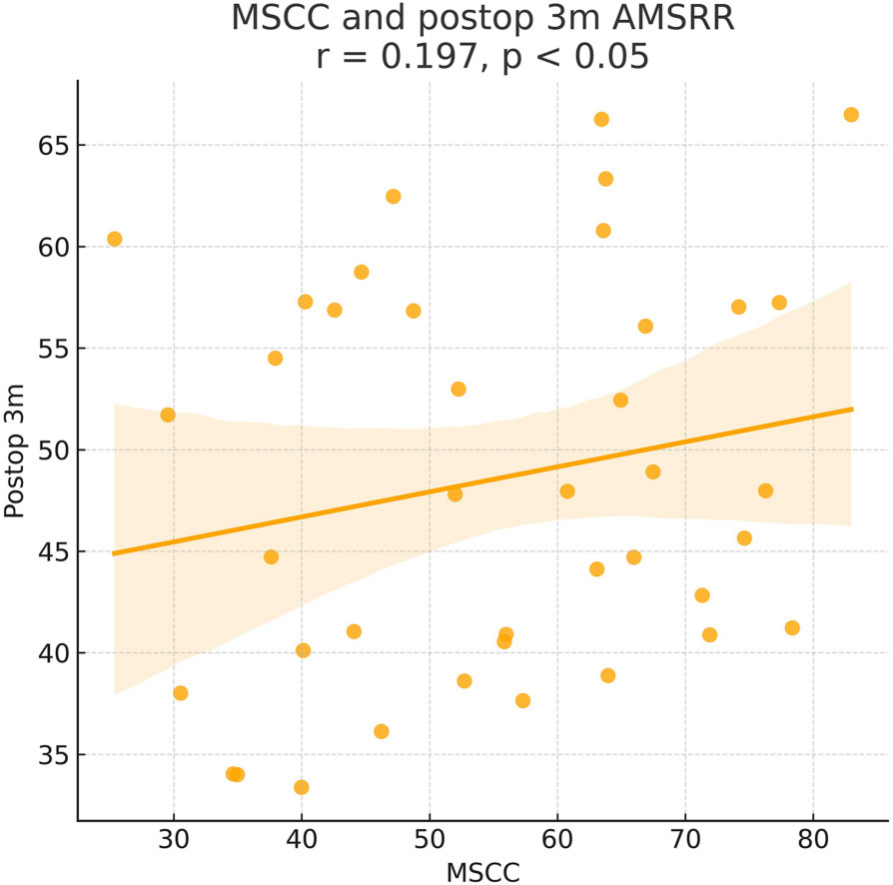
Pearson correlation analysis indicated a significant positive correlation between MSCC and 3-month postoperative ASIA motor score recovery rate (AMSRR) (*γ* = 0.197, *P* < 0.05).

**Figure 7:**
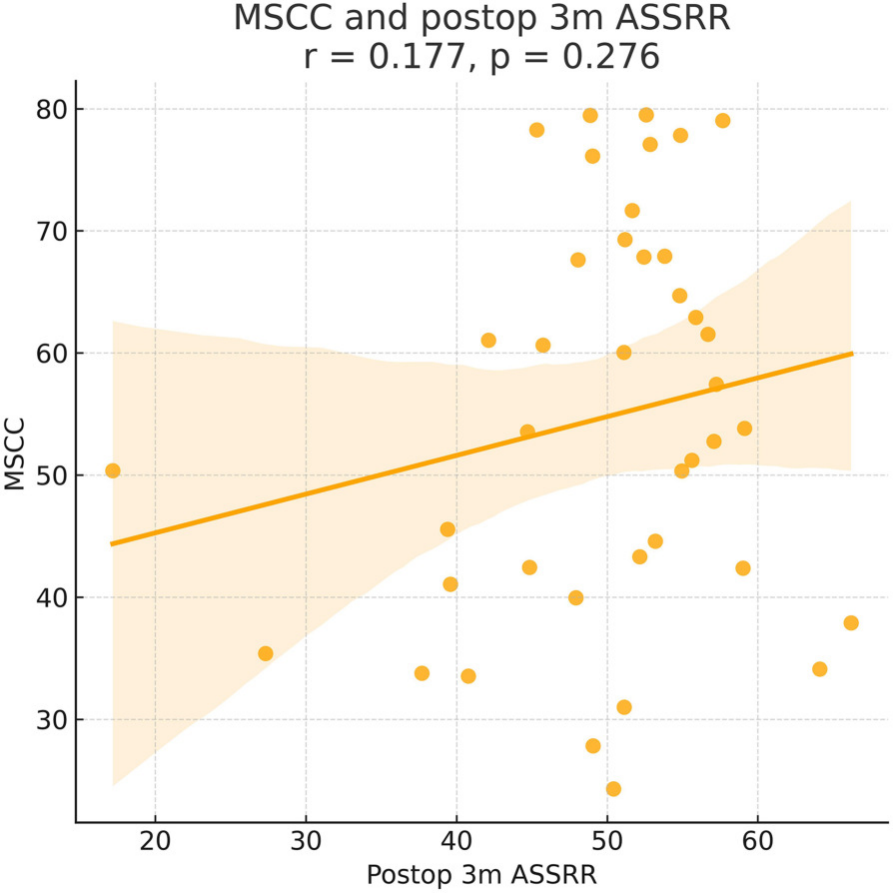
Pearson correlation analysis suggested a positive trend between MSCC and 3-month postoperative ASIA sensory score recovery rate (ASSRR), but the correlation was not statistically significant (*γ* = 0.177, *P* > 0.05).

As shown in the correlation matrix (Figures 4–7), MSCC demonstrated significant positive correlations with postoperative neurological recovery parameters, including RR, JOA, AMS, and AMSRR. This indicates that greater spinal cord compression may be associated with greater recovery potential, particularly in motor function. The correlation between MSCC and ASSRR was weaker, supporting the notion that sensory function recovery may be more delayed and less predictable. No significant correlation was observed between MSCC and age, gender, or surgical method, whereas a mild positive correlation with surgical timing suggests that patients with more severe compression may have undergone earlier intervention.

### 3.2 General, surgical, and MRI indicators

There were no statistically significant differences between the early and delayed surgery groups in terms of patient satisfaction at 2 years post-treatment, length of hospital stay, time to return to work, or hospitalization costs (*P* > 0.05). Although the early surgery group showed greater intraoperative complexity—such as longer operative duration, increased intraoperative blood loss, and higher postoperative drainage volume—compared with the delayed surgery group, the differences in surgical approach, number of fused levels, operative duration, intraoperative blood loss, postoperative complications, postoperative drainage volume, and bone fusion rate at 6 months were not statistically significant (*P* > 0.05). Regarding imaging parameters, both MCC and MSCC were higher in the early surgery group than in the delayed surgery group. However, only the difference in MSCC reached statistical significance (*P* < 0.05), while the difference in MCC did not (*P* > 0.05). Detailed results are presented in Table 1, and representative pre- and postoperative imaging findings, along with MSCC values, are shown in Figures 2, 3.

### 3.3 Efficacy evaluation

**Figure 8:**
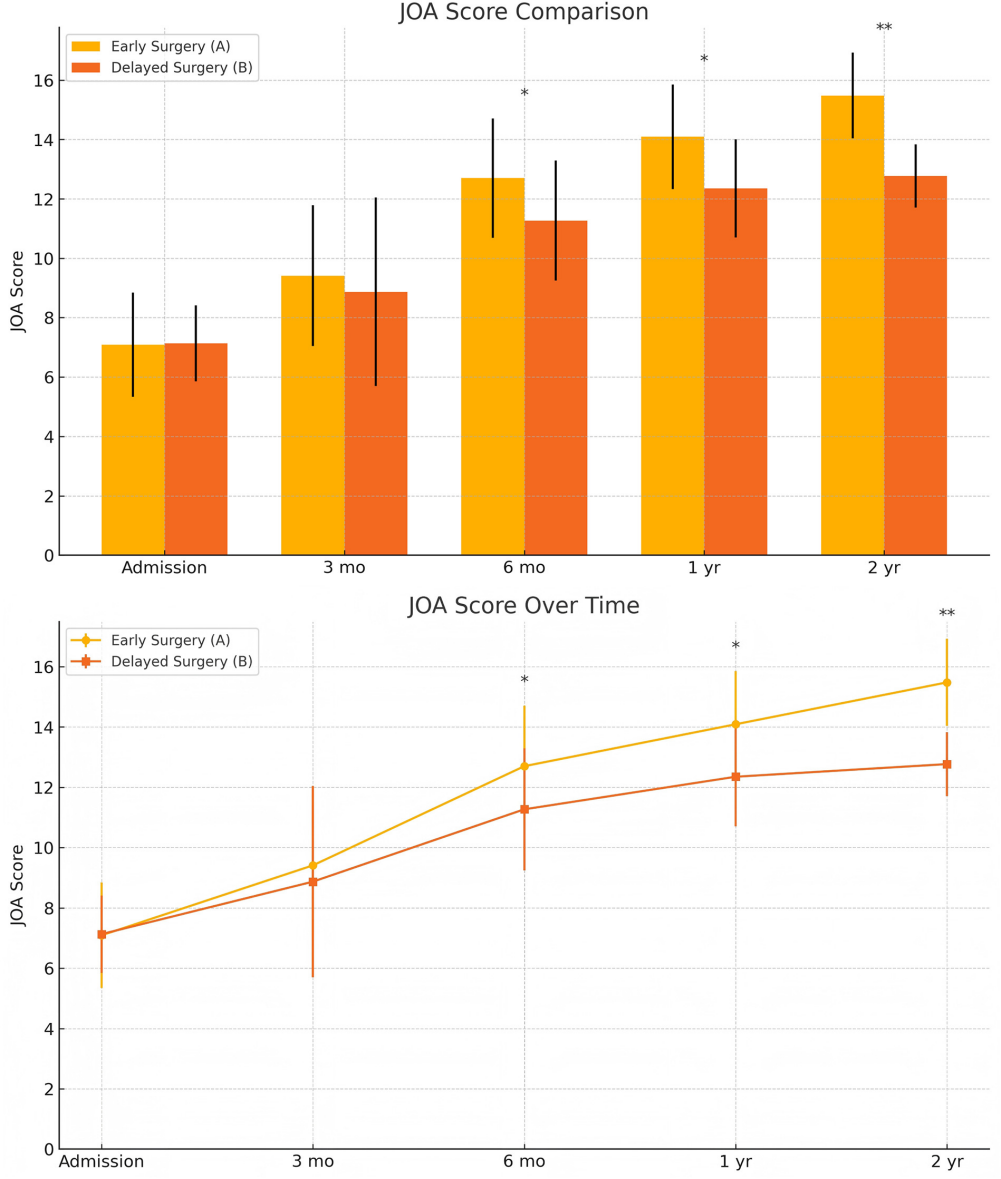
Changes in JOA scores at admission and postoperatively between the two groups.

**Figure 9:**
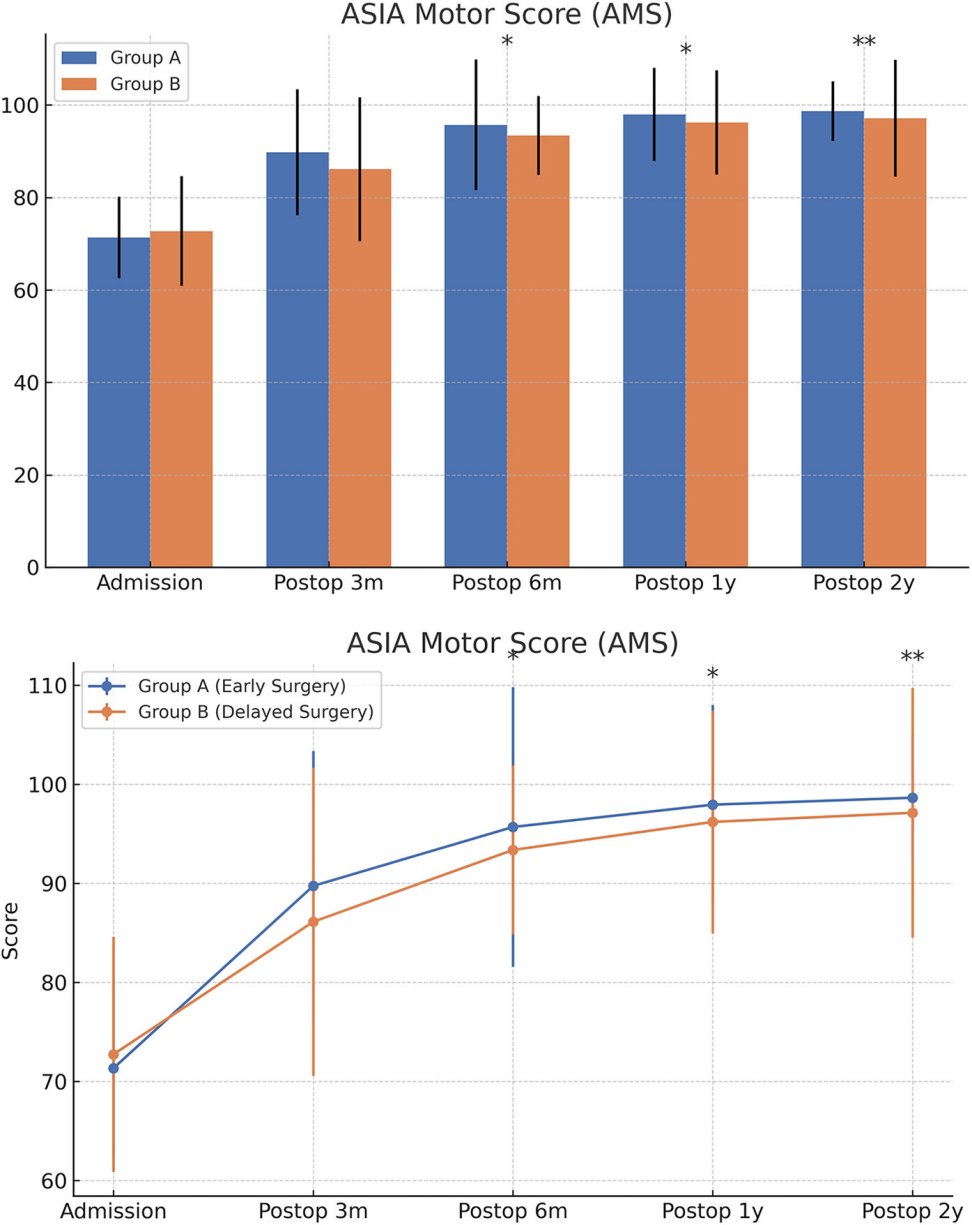
Changes in ASIA motor scores (AMS) at admission and postoperatively between the two groups.

**Figure 10:**
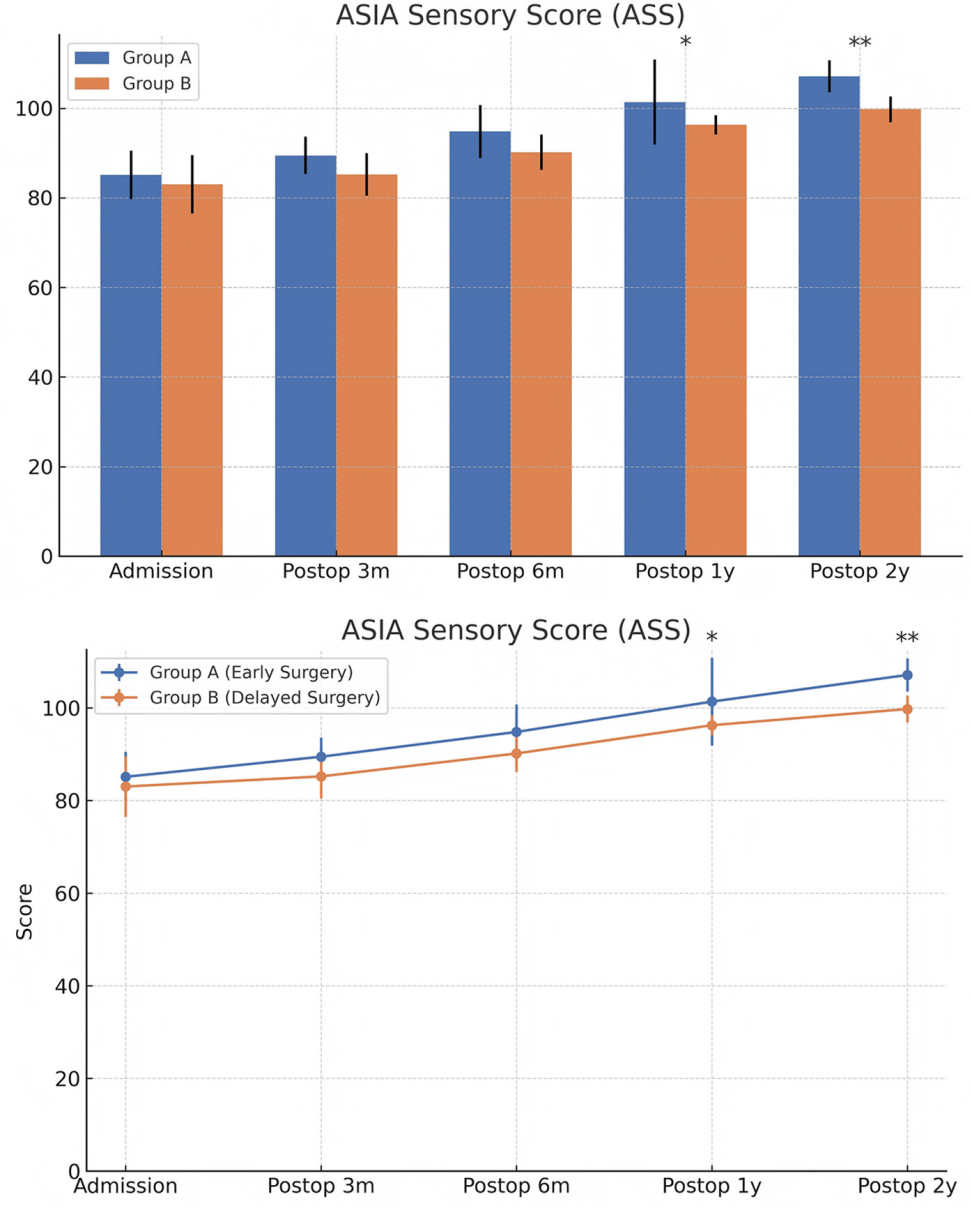
Changes in ASIA sensory scores (ASS) at admission and postoperatively between the two groups. The score comparison charts in Figure 4 include error bars and significance markers. Error bars represent standard deviations, indicating the variability within each group. Significance markers use asterisks (*, **, ***) to denote levels of statistical significance: * indicates *P* < 0.05;** indicates *P* < 0.01;*** indicates *P* < 0.001; lack of an asterisk indicates no statistically significant difference.

At admission, there were no significant differences between the two groups in terms of JOA score, AMS, or ASS. Following surgical treatment, all neurological function scores improved at follow-up compared to baseline in both groups, with the early surgery group consistently outperforming the delayed surgery group. At 6 months, 1 year, and 2 years postoperatively, JOA and AMS scores showed statistically significant intergroup differences (*P* < 0.05), while ASS scores showed significant differences at 1 and 2 years (*P* < 0.05). Notably, the difference in JOA scores at 2 years post-treatment was 2.71 points, exceeding the minimum clinically important difference (MCID). Regarding recovery rates, RR, ASSRR, and AMSRR were all significantly higher in the early surgery group at both 1 and 2 years postoperatively (*P* < 0.05). In the early surgery group, Pearson correlation analysis revealed that MSCC was positively correlated with postoperative 3-month RR (*γ* = 0.527, *P* < 0.05) and AMSRR (*γ* = 0.197, *P* < 0.05). Although a weak positive correlation was observed between MSCC and postoperative 3-month ASSRR (*γ* = 0.177), the association was not statistically significant (*P* > 0.05). Detailed results are presented in Figures 8–10; Table 2.

**Table 2:** Comparison between the two sets of observational indicators and evaluation criteria.

Variable	Early surgery group Group A	Delayed surgery group Group B	*P* score
JOA score			
Admission	7.09 ± 1.75	7.13 ± 1.28	0.437
Postoperative 3 month	9.41 ± 2.37	8.87 ± 3.17	0.292
Postoperative 6 month	12.70 ± 2.01	11.27 ± 2.02	0.038
Postoperative 1 years	14.09 ± 1.76	12.35 ± 1.65	0.022
Postoperative 2 years	15.48 ± 1.44	12.77 ± 1.06	0.009
JOA score recovery rate (RR) (%)			
Postoperative 3 month	28.46 ± 8.49	17.63 ± 18	0.384
Postoperative 6 month	56.61 ± 9.44	41.95 ± 2.89	0.153
Postoperative 1 years	70.64 ± 5.72	52.89 ± 7.54	0.014
Postoperative 2 years	84.66 ± 2.19	57.14 ± 2.86	0.007
ASIA motor score (AMS)			
Admission	71.35 ± 8.84	72.75 ± 11.86	0.643
Postoperative 3 month	89.75 ± 13.62	86.13 ± 15.56	0.288
Postoperative 6 month	95.70 ± 14.10	93.38 ± 8.86	0.037
Postoperative 1 years	97.95 ± 10.05	96.21 ± 14.52	0.011
Postoperative 2 years	98.65 ± 6.46	97.13 ± 12.59	0.005
ASIA motor score recovery rate (AMSRR) (%)			
Postoperative 3 month	64.22 ± 3.39	49.11 ± 9.17	0.425
Postoperative 6 month	84.99 ± 1.27	75.71 ± 6.42	0.171
Postoperative 1 years	92.84 ± 4.68	86.09 ± 1.74	0.002
Postoperative 2 years	95.28 ± 7.96	89.47 ± 7.89	0.000
ASIA sensory score (ASS)			
Admission	85.12 ± 5.40	83.01 ± 6.52	0.588
Postoperative 3 month	89.45 ± 4.18	85.20 ± 4.74	0.274
Postoperative 6 month	94.80 ± 5.93	90.15 ± 3.96	0.197
Postoperative 1 years	101.35 ± 4.95	93.30 ± 2.94	0.021
Postoperative 2 years	107.10 ± 3.57	96.25 ± 2.19	0.004
ASIA sensory score recovery rate (ASSRR) (%)			
Postoperative 3 month	35.20 ± 20.10	24.95 ± 10.11	0.233
Postoperative 6 month	58.70 ± 17.04	35.45 ± 13.28	0.618
Postoperative 1 years	80.40 ± 14.83	45.10 ± 12.82	0.017
Postoperative 2 years	85.10 ± 14.69	57.90 ± 18.55	0.000
General indicator			
Patient satisfaction (%)	83.58 ± 9.38	79.19 ± 8.955	0.073
Hospital stays (days)	9.63 ± 5.95	10.33 ± 3.622	0.093
Treatment cost (yuan)	68,217.90 ± 1,187.04	72,990.91 ± 1,217.42	0.125
Resume working times (days)	7.62 ± 2.36	8.05 ± 1.932	0.317

## 4 Discussion

Cervical spinal cord injury without fracture or dislocation (CSCIwoFD) is a common type of cervical spinal cord injury in spinal surgery, and the optimal timing of surgical intervention has long been a focal point of clinical discussion. However, the most appropriate timing for surgery remains controversial. Most previous studies have used 24 or 48 h post-injury as the cutoff for early vs. delayed surgery (4, 11), focusing primarily on the comparison of neurological recovery outcomes. Only a few studies have explored the therapeutic efficacy of delayed surgical intervention (6). In considering surgical timing, we accounted for the following factors: in the acute phase of spinal cord injury, hemorrhage and edema are predominant pathological features; in some cases, cervical trauma leads to spinal cord concussion, which clinically mimics CSCIwoFD. Cervical spine T2-weighted MRI images during the acute phase commonly exhibit high signal intensity due to spinal cord edema, which increases the risk of misdiagnosis. From the perspective of MRI-based classification, edema-type injuries typically present with high signal intensity on T2-weighted images, whereas hemorrhagic-type injuries may also demonstrate high signal intensity on T2-weighted imaging approximately 7 days post-injury (12). Therefore, we adopted a 7-day postoperative interval as the threshold to divide patients diagnosed with CSCIwoFD and treated surgically at our institution into two groups: the early surgery group (Group A, ≤7 days post-injury) and the delayed surgery group (Group B, >7 days post-injury). This study aims to investigate and compare the therapeutic efficacy of early vs. delayed surgery for CSCIwoFD, in order to provide evidence-based guidance for optimal surgical timing in future clinical practice.

In this study, we conducted long-term follow-up of patients with cervical spinal cord injury without fracture or dislocation (CSCIwoFD) to evaluate the long-term neurological recovery following surgical treatment. Neurological function was assessed using the Japanese Orthopaedic Association (JOA) score, ASIA sensory score (ASS), and ASIA motor score (AMS), recorded at baseline and at 3 months, 6 months, 1 year, and 2 years post-treatment. Corresponding improvement rates were also calculated. Radiographic parameters included the assessment of developmental spinal canal stenosis and ossification of the posterior longitudinal ligament. Quantitative imaging indicators such as the maximum canal compromise (MCC) and the maximum spinal cord compression (MSCC) were measured. These imaging findings were statistically analyzed in relation to postoperative neurological scores and their improvement rates using R software, including linear correlation analysis and multivariate regression analysis.

Pearson correlation analysis first revealed that in the early surgery group, the degree of spinal cord compression (MSCC) was significantly positively correlated with postoperative neurological recovery parameters, including RR, JOA score, AMS, and AMSRR. Specifically, MSCC showed a statistically significant positive correlation with RR and AMSRR at 3 months postoperatively, suggesting that a greater degree of spinal cord compression may be associated with a higher potential for motor function recovery. This finding indicates that MSCC may serve as a predictive marker for early postoperative motor function recovery. The correlation between MSCC and the ASSRR was weaker. Although a positive trend was observed between MSCC and ASSRR at 3 months, it did not reach statistical significance, supporting the notion that sensory function recovery is generally more delayed and less predictable than motor recovery. No significant correlations were observed between MSCC and patient age, sex, or surgical approach. A mild positive correlation was noted between MSCC and surgical timing. When comparing groups, both the early and delayed surgery groups demonstrated postoperative improvements in JOA score, ASS, and AMS relative to preoperative values. However, the early surgery group showed significantly better outcomes. Statistically significant differences (*P* < 0.05) were observed between the groups in JOA and AMS scores at 6 months, 1 year, and 2 years postoperatively, as well as in ASS scores at 1 and 2 years. Notably, the difference in JOA scores between the two groups at 2 years post-treatment was 2.71 points, exceeding the minimum clinically important difference (MCID) of 2.5 points for the JOA scale (13). Regarding improvement rates, statistically significant differences (*P* < 0.05) were found between the groups in RR, ASSRR, and AMSRR at 1 and 2 years postoperatively. These findings indicate that early surgical intervention results in more favorable neurological functional recovery compared to delayed surgery.

In recent years, with the gradual development of society, the incidence of spinal cord injury (SCI) has been on the rise. Cervical spinal cord injury without fracture or dislocation (CSCIwoFD) represents a distinct subtype of cervical SCI. Studies have reported that SCI accounts for approximately 0.2%–0.5% of all traumatic injuries, while CSCIwoFD comprises about 37%–52% of cervical spinal cord injuries (14). In adults, CSCIwoFD predominantly affects the cervical spine. The most common injury mechanisms include hyperextension, flexion, and traction of the head and neck (15, 16). CSCIwoFD is typically characterized by a short segment of injury, predominantly incomplete SCI, and severe sensory deficits below the affected level (17). In cases where complete SCI occurs, the prognosis is generally poor. Numerous studies have suggested that a history of cervical spinal canal stenosis—caused by conditions such as cervical disc herniation, ossification of the posterior longitudinal ligament, or ligamentum flavum calcification—is closely associated with the pathogenesis of CSCIwoFD. External trauma further compromises the already limited canal reserve capacity, resulting in spinal cord compression. This aligns with findings on post-injury MRI (18, 19). Samsani et al. (20) proposed that spinal cord trauma induces hemodynamic disturbances, leading to hemorrhage and edema, which further exacerbate spinal cord injury and contribute to vascular damage, forming a vicious cycle. This phenomenon is also reflected in MRI-based cervical spinal cord injury classifications (12). The presence of hemorrhage, edema, and tissue damage manifests differently on T2-weighted images, and these imaging subtypes may influence decisions regarding surgical timing, operative strategy, and prognosis.

Although the role of surgical intervention in the treatment of CSCIwoFD remains controversial, decompression and fusion procedures should be considered in patients with clinical and MRI evidence of persistent spinal cord compression and instability. Previous research conducted at our institution comparing conservative treatment and surgical intervention for CSCIwoFD demonstrated that both ACDF and conservative treatment were effective in patients with MRI-confirmed spinal cord compression. However, surgical treatment, particularly ACDF, was associated with superior clinical outcomes and better neurological recovery, indicating that ACDF may offer greater therapeutic benefits for patients with CSCIwoFD. For patients whose primary injury involves one to two cervical levels, the surgical approach is predominantly anterior. ACDF is the most commonly employed anterior technique and has been validated in prior studies as a feasible treatment for CSCIwoFD (21). In contrast, anterior cervical corpectomy decompression and fusion (ACCF) is a less frequently utilized procedure with limited indications and technical data. Due to its rare application in our practice, ACCF was not included in the current study. Posterior cervical surgery is more appropriate for cases involving multilevel degeneration (more than two levels), particularly when spinal cord injury is accompanied by extensive ossification of spinal ligaments. In such scenarios, posterior approaches allow for direct facet joint release and thorough decompression of the posterior spinal canal, offering enhanced stabilization for multilevel spinal cord injuries. Anterior cervical procedures enable direct visualization and removal of compressive disc material, either under direct vision or with microscopic assistance, thereby relieving spinal cord compression, reconstructing cervical alignment, and stabilizing the vertebral column. These procedures are associated with relatively simple surgical access, lower intraoperative blood loss, and facilitate postoperative rehabilitation and functional recovery. On the other hand, posterior cervical surgery is advantageous in resolving posterior compressive pathologies and provides superior stability in multilevel injuries. In this study, the surgical approach was not identified as a significant factor influencing spinal cord functional scores or improvement rates. This finding suggests that both anterior and posterior surgical approaches can achieve adequate spinal cord decompression and stabilization, thereby promoting neurological recovery. The choice of surgical method should therefore be based on the number, location, and characteristics of the injured segments, and it is unlikely to affect the neurological outcomes associated with different surgical timing.

For the purpose of research, the neuropathological changes in the spinal cord following spinal cord injury (SCI) are generally divided into three stages (22, 23): (1) the early inflammatory response phase, occurring within 3 days post-injury; (2) the necrotic debris clearance phase, from day 4 to 2 weeks post-injury; and (3) the astrocyte proliferation phase, occurring beyond 2 weeks post-injury. During the early inflammatory phase, the injured spinal cord segment typically exhibits edema and hemorrhage, which appear as hyperintense and hypointense signals, respectively, on T2-weighted MRI. These findings have proven diagnostic value in cases of SCIWORA (24). However, it remains difficult to distinguish between spinal cord injury and spinal cord concussion on early MRI images. Spinal cord concussion generally resolves with conservative treatment, with symptoms such as numbness and pain typically disappearing within 3 days. Persistent neurological symptoms beyond this period often indicate true spinal cord injury. In the astrocyte proliferation phase, excessive activation and proliferation of astrocytes result in the formation of glial scars, which inhibit axonal regeneration (25). Prolonged compression of the spinal cord or nerves can thus lead to irreversible damage. Given these observations, the necrotic debris clearance phase is likely the critical window for effective intervention in SCI. Previous studies have shown that specific RNAs, such as miRNA-133b, play a regulatory role in SCI, with expression beginning to increase 4–6 h post-injury and peaking significantly at 24 h and 7 days (26). Research by Kigerl et al. (27) demonstrated that both M1 (CD16/32^+^) and M2 (arginase-1^+^) phenotypes of microglia can be observed within one week in a mouse SCI model. However, only M1-type microglia persist until day 28 post-injury. iNOS-positive M1 microglia peak in expression at day 1, while arginase-1-positive M2 microglia show increased expression between days 4 and 7 post-injury (22). Additional studies have suggested that during the cellular regulation process following SCI, changes in the expression and activity of phosphorylated STAT3 (pSTAT3) are involved in cellular hypertrophy and glial scar formation, with its activity peaking and declining primarily between 7 and 14 days post-injury (28). Based on these findings, we believe that the cellular responses and symptomatic manifestations in the subacute and chronic phases of cervical spinal cord injury also merit close investigation. The acute phase is marked by complex and potentially life-threatening hemorrhage and edema. In contrast, during the subacute phase, cellular responses involved in injury repair are more active, robustly expressed, and functionally significant, occurring in a hemodynamically more stable environment. Once chronic-phase scar tissue has formed, it represents additional damage to the spinal cord and occurs in a biologically inactive context that limits the potential for further repair. Consequently, the surgical risks and therapeutic efficacy vary across these different phases. Delaying surgery until the patient's overall health condition stabilizes may better support spinal segment stability and neurological recovery. While most previous studies and meta-analyses have focused on the outcomes of surgery performed within 24 or 72 h post-injury, only a few have addressed the efficacy of delayed surgical intervention (6). Therefore, in this study, we selected the 7-day post-injury mark as a threshold to investigate outcomes specifically during the necrotic debris clearance phase. We categorized CSCIwoFD patients undergoing surgical treatment at our institution into two groups: early surgery group (≤7 days) and delayed surgery group (>7 days), in order to evaluate and compare the therapeutic efficacy between the two timeframes.

In this study, we conducted long-term follow-up of patients with CSCIwoFD who underwent either early or delayed surgical treatment. We compared the JOA score, ASIA motor score (AMS), ASIA sensory score (ASS), and corresponding improvement rates (AMSRR and ASSRR) at admission and at 3 months, 6 months, 1 year, and 2 years postoperatively to evaluate neurological recovery after surgery. Within-group analysis: In both the early and delayed surgery groups, patients demonstrated improvements in JOA, AMS, and ASS scores during postoperative follow-up compared to their preoperative values. These findings suggest that neurological function improved regardless of whether surgery was performed early or later. Recovery tended to be more rapid within the first 6 months post-treatment, while the pace of improvement slowed thereafter. This indicates that spinal cord and neurological recovery occurs more rapidly in the short term, whereas long-term functional improvement is more gradual—findings that are generally consistent with previous studies (29). The MSCC was positively correlated with the RR at 3 months postoperatively (*γ* = 0.414, *P* < 0.05), and with AMSRR at 3 months (*γ* = 0.277, *P* < 0.05). Overall, MSCC was found to be positively associated with postoperative neurological recovery, especially with RR and AMSRR at 3 months, with correlation coefficients of 0.527 and 0.197, respectively. Between-group analysis: Among the general indicators, only the difference in patient satisfaction at 2 years post-treatment reached statistical significance (*P* < 0.05), while differences in other general data were not statistically significant (*P* > 0.05). Similarly, no significant differences were found between the groups regarding surgery-related parameters (*P* > 0.05). In terms of treatment efficacy, all neurological scores indicated better outcomes in the early surgery group compared to the delayed group, with statistically significant differences (*P* < 0.05). These results suggest that early decompression and internal fixation leads to superior neurological recovery, consistent with prior research findings (30). Regarding improvement rates, statistically significant differences were observed between the two groups in ASSRR and AMSRR at 2 years postoperatively (*P* < 0.05).

Having established that early surgical intervention yields better outcomes than delayed surgery, we further investigated the specific preoperative factors that may influence postoperative prognosis. Pearson correlation analysis was performed on various preoperative indicators. In the early surgery group, the degree of spinal cord compression (MSCC) was positively correlated with the 3-month postoperative RR and AMSRR, with correlation coefficients of 0.527 and 0.197, respectively. These findings suggest that the greater the degree of spinal cord compression, the more pronounced the functional improvement following surgery. This may be attributed to a stronger capacity for neurological recovery once decompression is achieved. The results support the notion that acute mechanical spinal cord compression, if promptly relieved, can lead to substantial neurological improvement—provided irreversible damage has not occurred. The observed correlation between MSCC and early postoperative recovery, particularly in motor function, implies that within a tolerable range, more severe compression may predict better short-term neurological recovery, especially in terms of muscle strength. This highlights the potential benefit of early surgical intervention in alleviating spinal cord compression and enhancing motor function recovery. At first glance, this finding may appear counterintuitive, as more severe compression is often presumed to result in worse outcomes. However, we hypothesize that patients with higher MSCC values in our cohort may have predominantly sustained acute mechanical compression without accompanying irreversible structural damage to the spinal cord. In such cases, although the spinal cord was significantly compressed, the underlying axonal integrity and functional pathways may have remained largely preserved. Prompt surgical decompression within a critical therapeutic window—such as the first 7 days post-injury—could have alleviated intramedullary pressure, restored spinal cord perfusion, and prevented secondary injury mechanisms, including ischemia, inflammation, and apoptosis. This would facilitate the recovery of previously suppressed but structurally intact neural pathways, resulting in a more pronounced functional improvement. This concept is supported by previous studies demonstrating that reversible spinal cord dysfunction—particularly in incomplete injuries—is amenable to timely surgical intervention before the onset of irreversible gliosis or axonal degeneration. Furthermore, the corticospinal tract, responsible for motor control, is especially vulnerable to compressive forces due to its superficial location within the spinal cord. Early decompression may prevent ongoing injury to this tract and preserve its capacity for conduction and plasticity. Therefore, in select patients with high MSCC but intact neurological substrates, early surgical intervention may act as a “rescue” mechanism, enabling rapid and meaningful neurological recovery. These findings underscore the need to consider both the quantitative severity and qualitative characteristics of spinal cord compression when evaluating surgical timing and prognosis.

In contrast, the correlation between MSCC and the recovery rate of ASSRR did not reach statistical significance. This may reflect the inherently delayed or less predictable trajectory of sensory pathway recovery compared to motor function. Sensory recovery is often more closely associated with long-term prognosis, and the current data may be insufficient to accurately predict its course. The lack of statistical significance could also be attributed to inter-individual variability and confounding factors such as age, baseline sensory function, and rehabilitation intensity. Therefore, larger-scale studies with longitudinal follow-up are warranted to further elucidate these relationships.

The potential underlying mechanisms for these findings are as follows: motor neurons are more susceptible to ischemia and mechanical compression. Spinal cord compression leads to a reduction in local blood flow, rendering motor neurons more vulnerable to ischemic injury. Early surgical intervention, by relieving compression and restoring perfusion, may significantly enhance the survival and functional recovery of motor neurons. The corticospinal tract, which is the principal pathway for motor function, is located in the superficial layers of the spinal cord and is therefore more prone to damage from external compression. Early decompression can mitigate ongoing injury to the corticospinal tract and facilitate the restoration of motor signal conduction. Compared with motor function, this study found that the predictive value of spinal cord compression severity (MSCC) for sensory function recovery was relatively limited. Sensory neurons are generally more resilient to ischemia and mechanical stress and may retain partial function even under compression. Although the correlation between sensory function recovery and the degree of compression is weaker, early surgical intervention may still indirectly promote sensory recovery by improving the local microenvironment and enhancing neuroplasticity. Clinically, preoperative assessment of MSCC may serve as an important predictor of postoperative motor function recovery. For patients with a higher degree of spinal cord compression, early surgical intervention may confer greater benefit and should be prioritized. The early phase following spinal cord injury represents a critical window for neurological recovery. Surgery performed within 7 days can relieve compression, restore blood perfusion, and significantly improve motor function outcomes. In addition, early intervention can reduce secondary injury processes such as inflammation, oxidative stress, and apoptosis, thereby creating a more favorable environment for neurological recovery. Moreover, early surgery can decrease the incidence of complications such as pulmonary infections, deep vein thrombosis, and pressure ulcers, further supporting patient recovery. In patients with high MSCC, the benefits of early surgical intervention are likely to be particularly pronounced.

In the management of patients with CSCIwoFD, early surgical intervention during the acute phase also carries certain disadvantages and remains a topic of ongoing debate. Although early surgery may facilitate decompression and spinal stabilization in some cases, its timing must be carefully considered. During the acute phase, the spinal cord is in a state of pronounced edema, congestion, and inflammation. The tissue is fragile, and intraoperative manipulation may exacerbate secondary spinal cord injury. Moreover, surgery during the acute phase is associated with higher anesthetic and intraoperative complication risks, including dural tears, intraoperative bleeding, postoperative infections, cerebrospinal fluid (CSF) leakage, and epidural hematoma. These complications may delay recovery and, in some cases, further aggravate neurological deficits. In the present study, a higher number of postoperative complications was observed in the early surgery group compared to the delayed surgery group. This may be attributed to the fact that most SCI patients present with high-energy traumatic events and often suffer from multiple or concomitant injuries. Although these patients may not have comorbidities such as cerebral infarction or intracranial hemorrhage, the acute traumatic and stress-related conditions are typically severe, with a higher incidence of hemorrhage and neural edema in the early post-injury period. Some studies have also suggested that in patients with acute traumatic spinal cord injury, vasogenic edema may not yet have peaked, and early decompressive surgery may not effectively reduce intramedullary pressure, potentially leading to progressive neurological deterioration (31). Under such circumstances, early surgery may require greater surgical precision and increased perioperative management efforts. Therefore, in the absence of clear evidence of progressive neurological deterioration or severe spinal cord compression, surgical intervention during the acute phase should be approached with caution.

In our study, although the incidence of postoperative complications was numerically higher in the early surgery group compared to the delayed group, the difference did not reach statistical significance. Nonetheless, it is important to examine the nature of these complications to fully assess the safety profile of early surgical intervention. The most frequently observed complications included urinary tract infections (UTIs), pneumonia, and cerebrospinal fluid (CSF) leakage. UTIs and pneumonia are common postoperative complications in spinal cord injury patients, particularly those with impaired mobility or bladder dysfunction. These were managed with appropriate antibiotic therapy and supportive care, and no cases resulted in sepsis or prolonged hospitalization. CSF leakage, while less common, occurred in a small number of patients and was successfully managed with conservative measures such as bed rest, hydration, and, in some cases, pressure dressings. None of the CSF leaks progressed to meningitis or required reoperation. Importantly, while these complications did not significantly delay neurological recovery, they underscore the need for heightened perioperative vigilance, especially in patients undergoing early surgery when tissue fragility and systemic stress responses may be more pronounced. The higher complication rate may reflect the physiological vulnerability of patients in the acute post-injury phase, during which inflammatory responses, edema, and coagulopathy are more active. Proactive perioperative care—including infection prevention protocols, early mobilization, and close monitoring—can help mitigate these risks and enhance recovery. Future prospective studies with larger sample sizes are warranted to better characterize the relationship between surgical timing and complication severity, and to develop optimized management strategies for high-risk patients.

In contrast, delayed surgery also offers certain clinical advantages. As the inflammatory response subsides and tissue conditions stabilize, the risk of intraoperative damage is reduced, thereby better preserving spinal cord function. Additionally, conservative management during the early phase allows for dynamic evaluation of neurological progression, which helps refine surgical indications and improve the precision of intervention. Delayed surgery also allows for more thorough preoperative evaluation, optimal surgical planning, and structured postoperative rehabilitation, thereby improving overall treatment efficiency. Furthermore, patients tend to have more stable psychological states during the delayed period, which can enhance postoperative compliance and rehabilitation outcomes. Although the incidence of complications between the early and delayed surgery groups did not differ significantly, this observation suggests that complications alone are not a decisive factor affecting outcomes. Nonetheless, the timing of surgery provides important insight into perioperative planning, intraoperative techniques, and postoperative symptom-targeted management.

## 5 Conclusion

This study demonstrates that both early and delayed surgical interventions can lead to improvements in spinal cord function in patients, with significantly greater improvement observed in those who underwent early surgery. From an imaging perspective, the maximum spinal cord compression (MSCC) was found to be a reliable predictor of motor function recovery in patients who received early surgical treatment. When determining the optimal timing for surgical intervention, it is essential to carefully weigh each patient's pre-existing comorbidities and the severity of injury. Once the acute traumatic stress response has stabilized, surgical intervention should ideally be performed within 7 days post-injury to maximize neurological recovery and improve overall prognosis.

## Data Availability

The original contributions presented in the study are included in the article/Supplementary Material, further inquiries can be directed to the corresponding author.
